# Thymoquinone Protective Effect Against Mercury-Induced Reproductive Derangement in Rats: In Vivo and In Silico Investigation

**DOI:** 10.3390/toxics13100896

**Published:** 2025-10-19

**Authors:** Solomon Owumi, Moses Otunla, Pelumi Akindipe, Uche Arunsi, Jesutosin O. Babalola, Chioma E. Irozuru, Ahmad Altayyar, Bayode Oluwawibe, Olatunde Owoeye, Adegboyega K. Oyelere

**Affiliations:** 1 Department of Biochemistry, University of Ibadan, Ibadan 200005, Oyo State, Nigeria; bosedepelu2014@gmail.com (P.A.); oluwawibe@gmail.com (B.O.); 2 Department of Pharmacology and Nutritional Sciences, College of Medicine, University of Kentucky, Lexington, KY 40536, USA; mtot223@uky.edu; 3 Department of Chemistry and Biochemistry, Georgia Institute of Technology, Atlanta, GA 30332, USA; venniabia@gmail.com (U.A.); adegboyega.oyelere@chemistry.gatech.edu (A.K.O.); 4 Nutrition and Industrial Biochemistry Research Laboratories, Department of Biochemistry, University of Ibadan, Ibadan 200005, Oyo State, Nigeria; jesutosinbabalola@gmail.com; 5 Department of Chemistry and Biochemistry, Montana State University, Bozeman, MT 59719, USA; chiomaejiro01@gmail.com; 6 Naz Coker Ovarian Cancer Research Centre (NOVARC), Nottingham Biodiscovery Institute, School of Medicine, University of Nottingham, Nottingham NG7 2RD, UK; ahmad.ty95@gmail.com; 7 Neuroanatomy Research Laboratories, Department of Anatomy, University of Ibadan, Ibadan 200005, Oyo State, Nigeria; oowoeye2001@yahoo.com

**Keywords:** thymoquinone, mercury chloride, reproductive toxicity, antioxidant, anti-inflammatory, apoptosis

## Abstract

Mercury exposure has been linked to male infertility. Given that mercury chloride (HgCl_2_) may promote an oxido-inflammatory milieu associated with pathophysiological derangements, it is hypothesised that Thymoquinone (TQ), an antioxidant and anti-inflammatory agent, may mitigate the gradual harmful effects of mercury exposure on rat testes, epididymis, and hypothalamus, as these organs are vital to reproductive function. To test this hypothesis, 40 rats (strain: Wistar; sex: male) were randomly assigned to five cohorts of eight rats each. After a 7-day acclimation, treatments were dispensed for 28 consecutive days accordingly: Cohort I: distilled water only, as control; Cohort II: HgCl_2_ only (20 µg/mL); Cohort III: TQ only (2.5 mg/kg); Cohort IV: HgCl_2_ + TQ (20 µg/mL + 2.5 mg/kg); and Cohort V: HgCl_2_ + TQ (20 µg/mL + 5 mg/kg). Co-treatment with TQ preserved the body and organ weight of the HgCl_2_ exposed animals. However, TQ did not reduce HgCl_2_-induced dysfunction in sperm function and morphology. The serum follicle-stimulating hormone (FSH), luteinising hormone (LH), and testosterone were increased significantly (*p* < 0.05) by TQ co-treatment, while decreasing the prolactin level. TQ administration also increased (*p* < 0.05) testicular enzymes, including alkaline phosphatase (ALP), lactate dehydrogenase (LDH), acid phosphatase (ACP), and glucose-6-phosphate dehydrogenase (G6PD) activities, which HgCl_2_ decreased. TQ administration increased (*p* < 0.05) HgCl_2_-induced decreases in catalase (CAT), superoxide dismutase (SOD), glutathione peroxidase (GPx), glutathione (GSH), glutathione-*s*-transferase (GST), and total sulfhydryl group (TSH) levels in the testes, epididymis, and hypothalamus of experimental rats. Further, TQ reduced HgCl_2_-mediated increases in RONS-reactive oxygen and nitrogen species; LPO–lipid peroxidation; PC–protein carbonyl formation; and XO–xanthine oxidase activity. Furthermore, levels of inflammatory biomarkers, including tumour necrosis factor alpha (TNF-α), nitric oxide (NO), interleukin-1 beta (IL-1β), and myeloperoxidase (MPO), were decreased (*p* < 0.05) in the co-treated groups, with a higher dose of TQ (5.0 mg/kg) showing a more pronounced protective effect. Additionally, TQ co-administration increased Bax and decreased Bcl-2 and p53 protein levels (*p* < 0.05), thereby protecting the rats’ testes, epididymis, and hypothalamus from HgCl_2_-induced apoptosis. Molecular docking simulation analysis revealed TQ interaction dynamics with PPAR-α and PPAR-δ to suppress NF-kB-mediated pro-inflammatory sequela as well as activate Nrf-2-mediated antioxidant defence system. These predicted biological effects of TQ resonate with the findings from the in vivo studies. Therefore, supplementation with TQ may help reduce chemical-induced toxicities, including HgCl_2_‘s reproductive toxicity.

## 1. Introduction

Mercury (Hg) is recognised globally as an environmental toxicant. Humans can be exposed to mercury in various forms: elemental mercury (Hg) through inhalation, inorganic mercury chloride (HgCl_2)_ through occupational or dental exposure, and organic methylmercury from consuming seafood. While HgCl_2_ does not cross the blood–brain barrier, it accumulates in reproductive organs, causing toxicity; it disrupts testicular architecture and inhibits spermatogenesis in rats [1]. Another study confirmed Hg-induced decreases in sperm production, increased DNA damage, and higher levels of reactive oxygen species (ROS) and lipid peroxidation in rat reproductive tissues [2]. Similarly, studies have revealed that HgCl_2_ increases malondialdehyde levels [3], the expression of tumour necrosis factor-α and cyclooxygenase-2 [4], and morphological changes in the reproductive tissues of male rats, including spermatogonium degeneration and thinning of the tubular wall in the testes [5]. Martinez et al. [1] emphasised that even at low doses, mercury impaired sperm membrane integrity, disrupted germinal epithelium, and increased ROS levels in male rats. Rats exposed to HgCl_2_ showed significantly reduced testosterone levels and nitric oxide production [6].

Additionally, the cellular antioxidant defence, represented by glutathione and enzymes like catalase, superoxide dismutase, and glutathione peroxidase, is significantly compromised upon treatment of experimental animals with HgCl_2_ [7]. Additionally, mercury exposure also disrupts cellular apoptotic balance [8]. For instance, apoptotic markers, such as Bax, were upregulated, indicating that cell death pathways were activated due to HgCl_2_ exposure. Given that experimental animals’ blood mercury levels were similar to those observed in humans after exposure [9], investigating protective agents against mercury toxicity becomes imperative.

Thymoquinone (TQ) is a bioactive compound from black seed oil (*Nigella sativa*) [3]. Studies have shown that TQ can mitigate reproductive toxicities in rats and improve reproductive parameters [10]. It decreases sperm DNA fragmentation (p53), modulates apoptosis-related genes, increasing Bax while reducing Bcl-2 [11]. TQ also counteracts lead acetate-induced reductions in sperm quality, testicular antioxidants, and reproductive hormone levels [12], highlighting its protective effects against a heavy metal [13,14]. Additionally, it modulates pathways crucial for cell survival and inflammation by downregulating TNF-α and NF-κB, thereby improving testicular histoarchitecture [15]. Given TQ’s multifaceted benefits, this study investigated its effects on HgCl_2_-induced reproductive toxicities in rats, utilising in vivo experiments and molecular docking studies. Our findings indicate that TQ supplementation reduces the adverse effects of HgCl_2_ on sperm function, prevents pro-oxidative and inflammatory pathophysiology, and improves cellular histology. Molecular docking simulations reveal TQ’s interaction with PPAR-α/β activates Nrf-2 and modulates NF-kB, promoting its beneficial antioxidant and anti-inflammatory effects.

## 2. Methods

### 2.1. Sample Size Determination and Animal Welfare

The study’s sample size was estimated using the G*Power Software version 3.1.9.445 [16]. Based on the effect size of 0.4 at *p*  <  0.05 for one-way analysis of variance (ANOVA), a total sample size of 125 is required with 95% power [17]. For the investigation, a group of forty male albino Wistar rats, with an average weight of 157 g, was obtained from the University of Ibadan. They were housed in standard cages at the animal facility of the University of Ibadan’s Biochemistry department. Before the study, the rats were acclimated to the study environment for seven days prior to study initiation and were maintained under consistent environmental conditions including temperature at 22 ± 2 °C, h light–dark cycle, with continuous access to food and water. All procedures adhered to the guidelines approved by the University of Ibadan Animal Care and Use Research Ethics Committee (UI-ACUREC), with approval number UI-ACUREC/057-1222/11.

We established inclusion and exclusion criteria a priori to ensure consistency and minimise bias. Animals were included if they met the following criteria, e.g., age range, weight range, and health status. They were excluded if they showed signs of unrelated illness, did not recover from anaesthesia, or demonstrated abnormal behaviour before treatment. To minimise potential confounders, treatments and measurements were performed in a randomised order to avoid systematic bias due to time-of-day or operator fatigue, cages were rotated weekly within the animal facility to mitigate location-based environmental effects (e.g., light, temperature, and noise), and investigators conducting outcome assessments were blinded to group allocation to reduce observer bias.

### 2.2. Chemicals

All chemicals and reagents used in this study were of the highest quality and were obtained from accredited commercial suppliers. See Supplementary Table S1 for a detailed list of the reagents, kits, and chemicals utilised for this study.

### 2.3. Experimental Protocol

**Figure 1:**
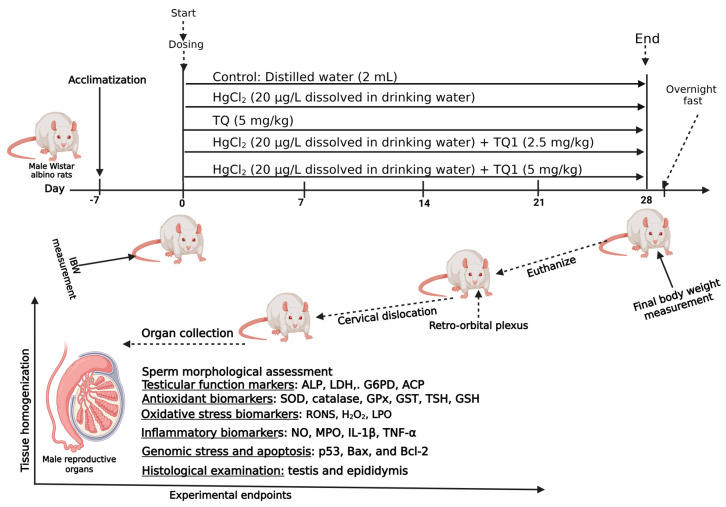
Experimental outlay to investigate Thymoquinone’s effect on mercuric chloride-treated Wistar albino rats (male) for 28 consecutive days. Created in Biorender. Uche Arunsi. (2025) https://BioRender.com.

Male Wistar rats (*n* = 40), ascertained to be healthy and free from physical deformities by a trained veterinarian, were randomly distributed into five experimental cohorts, each consisting of eight animals and were treated as inficated below and depicted in Figure 1.

Group 1: Control cohort (allowed free access to distilled water daily).

Group 2: HgCl_2_-only cohort: (20 µg/L HgCl_2_ dissolved in the drinking water).

Group 3: TQ-only cohort: (TQ 5 mg/kg bw per os/day).

Group 4: HgCl_2_ + TQ (Low dose TQ) cohort: (20 µg/L HgCl_2_ dissolved in drinking water + TQ 2.5 mg/kg bw per os/day).

Group 5: HgCl_2_ + TQ-treated rat (High dose) cohort (20 µg/L of HgCl_2_ dissolved in drinking water + TQ 5 mg/kg bw per os/day).

The doses were chosen based on previous studies from the scientific literature [10,18]. 20 µg/L of HgCl_2_ provided ad libitum was prepared by dissolving 10 g of HgCl_2_ in distilled water (100 mL), to yield a stock concentration (100 mg/mL). From the stock solution, 1 µL was further diluted in 5 L of distilled water, resulting in 20 µg/L of HgCl_2_ provided to the experimental rat via drinking bottles. Based on an estimated water intake of 9–12 mL per 100 g b.w, a rat of 200 g ingested approximately 0.36–0.48 µg/day, corresponding to 1.8–2.4 µg/kg/day, calculated using the formula below.

$$Intake\;\left(µg/kg/day\right)=\frac{Water\;intake\left(mL/day\right)\times Dose\;\left(µg/mL\right)}{Weight\;(kg)}\;$$

The substances were administered to non-fasted rats in the morning (between 09:00 and 10:00 h). The first day of treatment was designated as Experimental Day 0. At the end of the 4th week of treatment, the experimental rats were sacrificed and dissected. The testis, epididymis, and hypothalamus were swiftly removed for biochemical and histological examinations.

### 2.4. Study Conclusion and Euthanasia

After the last treatment with TQ and HgCl_2_, the rats were weighed, and their final body weights were recorded. Twenty-four hours later, blood samples were obtained from the left venous plexus using the retro-orbital method into plain tubes before euthanasia through cervical dislocation after carbon dioxide anaesthesia during tissue harvest, see Supplementary Materials (S2) for detailed method. The samples were stored at −80 °C after washing until required for biochemical analysis. The tissues were later homogenised using a Teflon homogeniser (Heidolph Silent Crusher M) in cold potassium phosphate buffer (0.1 M, pH 7.4). The protein concentration in the supernatant, obtained following centrifugation of the homogenate, was measured using the Lowry et al. method [19], with bovine serum albumin (BSA) serving as the standard.

### 2.5. Determination of Spermatozoa Motility

Spermatozoa progressive motility was evaluated according to established methodology [20]. Sperm were harvested from the cauda epididymis by incision and released onto a clean, sterilised slide. The sample was diluted with sodium citrate dehydrate solution, mixed gently, and covered with a coverslip for analysis. Sperm motility was assessed by analysing a minimum of ten microscopic fields using a phase contrast microscope (magnification: ×200). Within each field, sperm were classified according to their motility as progressive, non-progressive, or immotile. The outcome was reported as the percentage of sperm exhibiting progressive motility.

### 2.6. Assessment of Epididymal Sperm Count

The epididymal sperm count procedure follows the standards established by WHO [21]. The cauda epididymis is macerated in saline, subsequently filtered through nylon mesh, and a 5 μL aliquot of the resulting suspension is combined with 95 μL of diluent. After dispensing 10 μL of the diluted sperm suspension onto a hemocytometer and permitting sedimentation for 5 min, sperm cells are enumerated using an improved Neubauer chamber (Deep 1/10 m; LABART, Munich, Germany) under light microscopy (magnification: ×400).

### 2.7. Assessment of Sperm Morphological Abnormalities and Viability Assay

Sperm viability and morphological abnormalities were assessed following Wells and Awa [22]. Clean slides were used to prepare sperm smears, and viability was evaluated with eosin, nigrosine, and sodium citrate solutions of 1, 5, and 3%, respectively. A minimum of 400 sperm cells per rat were analysed for morphological abnormalities using a staining technique with 0.2 g eosin and 0.6 g fast green, prepared in a distilled water and ethanol solution (2:1 ratio). The proportion of head, midpiece, and tail abnormalities was systematically documented for both control and treated groups.

### 2.8. Assessment of Reproductive Hormone Levels

Serum levels of luteinizing hormone (LH), follicle-stimulating hormone (FSH), prolactin, and testosterone were measured using ELISA kits from Elabscience Biotechnology (Wuhan, China), following the manufacturer’s protocols. The detection limits for the assays were 0.54 ng/mL for LH, 0.28 ng/mL for FSH, 0.39 ng/mL for prolactin, and 0.58 ng/mL for testosterone. All hormone measurements were conducted simultaneously to reduce inter-assay variability, with intra-assay coefficients of variation recorded at 2.9% for FSH, 3.3% for LH, 2.4% for prolactin, and 3.8% for testosterone.

### 2.9. Assessment of Testicular Enzyme Function

The activities of acid and alkaline phosphatase (ACP and ALP) in the testicular supernatant were measured according to established protocols, utilising p-nitrophenyl phosphate hydrolysis under acidic [23] and alkaline [24] conditions. Testicular glucose-6-phosphate dehydrogenase (G6PD) activity was determined using NADP+ and glucose-6-phosphate as substrates, employing a modified procedure adapted from Wolf et al. [25]. Lactate dehydrogenase-X (LDH-X) activity was analysed following Vassault’s protocol [26,27], which assesses the interconversion between pyruvate and lactate.

### 2.10. Evaluation of Testes, Epididymides, and Hypothalamus Antioxidant Biomarker Status

Testes, epididymides, and hypothalamus homogenates were used for the assessment of oxidative stress, inflammation, and apoptosis. Superoxide dismutase (SOD) [28] and catalase (CAT) [29] were assessed using the referenced biochemical methods. Enzyme activities for glutathione-*s*-transferase (GST) [28] and glutathione peroxidase (GPx) [25] were also determined biochemically. Reduced glutathione (GSH) was quantified according to the Beutler method [30], while total sulfhydryl groups (TSH) were quantified by the method of Jollow et al. [31]; see Supplementary Materials (S2) for the detailed methods. Furthermore, key biomarkers indicative of cellular responses to oxidative stress—namely NRF-2, HO-1, as well as TRX concentrations and TRX-R enzymatic activity—in the testes, epididymis, and hypothalamus of the experimental animals, were measured using rat-specific enzyme-linked immunosorbent assay (ELISA) kits, adhering to the manufacturer’s instructions as outlined in previous reports [32,33].

### 2.11. Assessment of RONS and LPO Concentrations and XO Activity in the Testes, Epididymides, and Hypothalamus of Rats

RONS-dependent oxidation of DCFH-DA-2′,7′-dichlorodihydrofluorescein diacetate- to DCF- dichlorofluorescein was employed to evaluate RONS in the testes, epididymis, and hypothalamus [33]. Lipid peroxidation was measured by assessing the formation of TBARS following the Okhawa method [34]. The method of Bergmeyer et al. [34] was used to assess the activity of xanthine oxidase (XO) in the examined samples. See Supplementary Materials (S2) for the detailed method.

### 2.12. Assessment of Pro-Inflammatory Markers in Rat Testes, Epididymides, and Hypothalamus

Nitric oxide (NO) concentration from the homogenised tissue supernatant was measured using Green’s method [35]. Myeloperoxidase (MPO), indicating polymorphonuclear leukocyte accumulation, was assessed with a modified Trush method [36], where MPO oxidises o-dianisidine with H_2_O_2_, producing a molecule detected at 470 nm. Testicular, epididymal, and hypothalamic IL-1 were quantified using ELISA kits per the manufacturer’s instructions.

### 2.13. Evaluation of Apoptosis Biomarkers

The concentrations of Bcl-2 Associated X Protein (Bax), protein 53 (TP53), and B-cell Lymphoma 2 (Bcl-2) were measured from the supernatant of the tissue homogenate using ELISA, according to the manufacturer’s instructions; see Supplementary Materials (S2) for detailed Method.

### 2.14. Histopathological Examination of the Testes, Epididymides, and Hypothalamus

Histopathological analysis of the testes, epididymides, and hypothalamus was conducted using standard and advanced histology tissue processing and microscopy techniques [37,38]. After euthanasia, the hypothalamus was harvested and treated with 10% formalin solution, while the harvested epididymides and testes (sliced through to enhance rapid penetration of the fixative) were quickly fixed in Bouin’s solution. See Supplementary Materials (S2) for the detailed method. For each group (*n* = 3 rats/group), three slides were prepared per rat for both testes and epididymis, followed by Hematoxylin and eosin staining. Each stained slide was imaged using a light microscope at five random, non-overlapping fields. The seminiferous tubular diameter and epididymal epithelial thickness were quantified with ImageJ software (version 1.50i; Wayne, Rasband, National Institute of Health, Bethesda, MD, USA, https://imagej.nih.gov/ij/, accessed on 14 October 2025) at 400× magnification.

### 2.15. Molecular Docking Method

Molecular docking was conducted to assess the interaction between TQ and PPAR-α or PPAR δ/β. The molecular docking scores, reflecting the binding affinity between the ligand and the proteins, were quantified as binding constants (Kd). To achieve this, the 3D structures of TQ were sourced from PubChem: https://pubchem.ncbi.nlm.nih.gov, the URL was accessed on 15 June 2025, while the structures of the PPAR-α (PDB: 1I7G) and PPAR- δ/β (PDB: 3D5F) were acquired from the Protein Database (PDB): https://www.rcsb.org/, the URL was accessed on 15 June 2025. Protein preparation was performed using UCSF ChimeraX [39]. See Supplementary Materials (SM) for the detailed method.

### 2.16. Statistical Analysis

Data analysis was performed using the Windows version of GraphPad Prism (www.graphpad.com; GraphPad, CA, USA; v.9.3.1). One-way analysis of variance (ANOVA) followed by Bonferroni post hoc testing was conducted to compare group means. Statistical significance was defined as *p* < 0.05. Results are presented as mean ± standard deviation (SD) of the replicates.

## 3. Results

### 3.1. The Effect of TQ on the Body Weight and Organosomatic Index of Experimental Animals Treated with HgCl_2_

Table 1 depicts TQ’s body weight and organ weight-conserving ability against HgCl_2_-treated experimental rats. HgCl_2_ exposure resulted in decreased body weight gain and alterations in the organ weight and relative weights of testes, epididymides, and hypothalamus compared to the control group. However, treatment with low and high doses of TQ (2.5 and 5.0 mg/kg) mitigated these effects induced by HgCl_2_ exposure, improving body weight gain and restoring the relative organ weights.

### 3.2. Effect of TQ on Sperm Functional Parameters and Morphological Abnormalities in HgCl_2_-Treated Rats

Table 2 indicates the effect of TQ on sperm functional parameters and morphological abnormalities in HgCl_2_-treated rats. Compared to the control, exposure to HgCl_2_ significantly reduced sperm motility. Interestingly, co-treatment with 2.5 and 5 mg/kg TQ failed to reverse these changes. Administration of rats with HgCl_2_ significantly decreased the sperm count from 132.4 million/mL in the control group to 117 million/mL in animals that received HgCl_2_ alone. At the same time, there were no changes in sperm volume across all groups. Furthermore, exposure of animals to HgCl_2_ induced morphological changes to the sperm, which were not reversed upon co-treatment with TQ.

### 3.3. TQ Improved HgCl_2_-Induced Alteration in Reproductive Hormones in the Serum of Treated Rats

**Figure 2:**
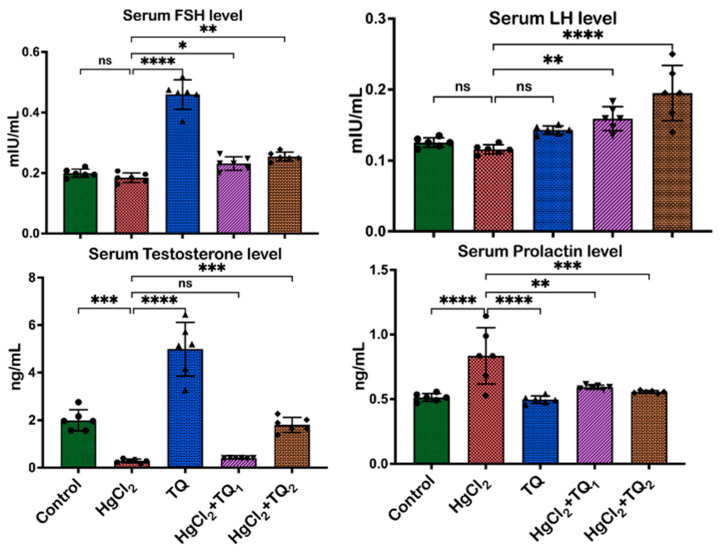
The effect of Thymoquinone on reproductive hormones in rats treated with mercuric chloride for 28 days. Results are shown as mean ± SD (*n* = 6), *p* < 0.05, 0.01, 0.001, 0.0001 (*, **, ***, ****), ns: not significant. FSH: follicle-stimulating hormone, and LH: luteinising hormone.

The impact of TQ co-treatment on the serum level of reproductive hormones in HgCl_2_-treated experimental rats is shown in Figure 2. Upon exposure to HgCl_2_ alone, there was a decrease in the serum levels of LH [F (4, 25) = 15.20, *p* < 0.0001], FSH [F (4, 25) = 107.9, *p* < 0.0001], and testosterone [F (4, 25) = 68.92, *p* < 0.0001] by 10.60%, 9.91%, and 84.69%, respectively, compared to the control group. Conversely, the serum prolactin level [F (4, 25) = 11.45, *p* < 0.0001] increased by 62.67% with HgCl_2_ treatment relative to the control. However, co-treatment with TQ (2.5 mg/kg) resulted in increases of 43.81%, 32.11%, and 30.36% in LH, FSH, and testosterone serum levels, respectively. Moreover, a 29.00% decrease in prolactin levels was observed in the TQ co-administered group (2.5 mg/kg). Furthermore, when compared to animals that received HgCl_2_ alone, 5.0 mg/kg of TQ co-treatment induced even more pronounced changes, as seen in 80.86%, 41.78%, and 515.08% increases in serum levels of LH, FSH, and testosterone, respectively, and a 33.43% decrease in prolactin.

### 3.4. TQ Co-Administration Increased the Activities of Testicular Enzymes in Experimental Animals

**Figure 3:**
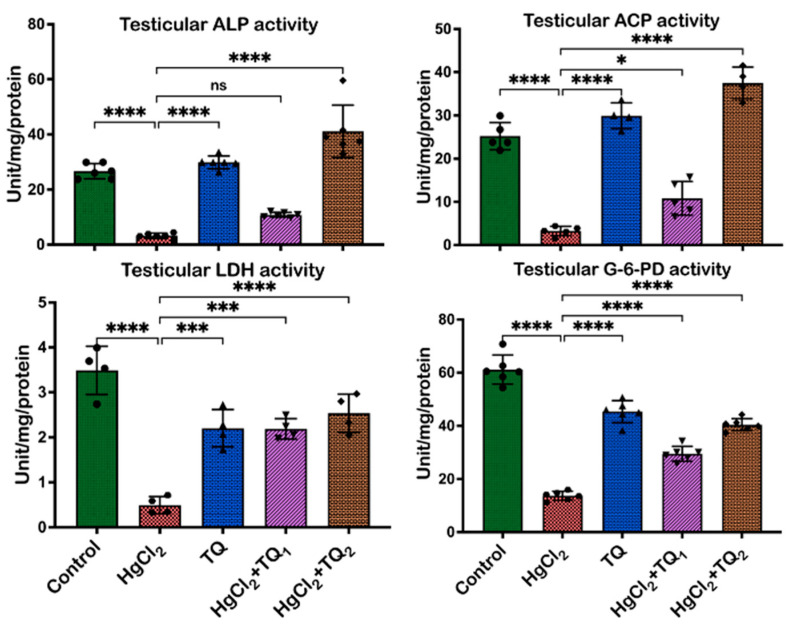
The effect of Thymoquinone on testicular enzyme activities in rats treated with mercuric chloride for 28 days. Results are shown as mean ± SD (*n* = 8), *p* < 0.05, 0.001, 0.0001 (*, ***, ****), ns: not significant. ALP: Alkaline phosphatase; ACP: Acid phosphatase; LDH: Lactate dehydrogenase; G6PD: Glucose-6-phosphate dehydrogenase.

Figure 3 displays the result of TQ co-administration on the activities of testicular enzymes in experimental animals exposed to HgCl_2_. Exposure of animals to HgCl_2_ alone significantly decreased the serum activities of ALP [F (4, 25) = 88.76, *p* < 0.0001], ACP [F (4, 25) = 56.62, *p* < 0.0001], LDH [F (4, 25) = 61.42, *p* < 0.0001] and G6PD [F (4, 25) = 150.2, *p* < 0.0001] by 87.53%, 87.53%, 85.85%, and 77.26%, respectively, compared to the control group. However, TQ administration increased the dose-dependent activities of testicular enzymes. Co-treatment with 2.5 mg/kg TQ significantly increased ALP and ACP serum activities by 233.33% each, LDH by 343.27%, and G6PD by 110.47% relative to the HgCl_2_-alone administered group. Furthermore, a higher dose of TQ co-treatment (5 mg/kg) led to even more significant increases in serum enzyme activities: ALP and ACP each by 1054.96%, LDH by 413.78%, and G6PD by 188.74% compared to the HgCl_2_ alone group.

### 3.5. TQ Co-Treatment Restored the Antioxidant Status of Rats Treated with HgCl_2_

**Figure 4:**
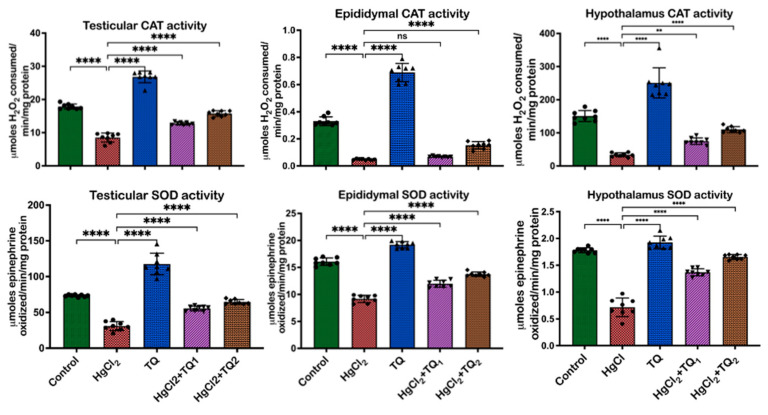
The effect of Thymoquinone (TQ) on catalase (CAT) and superoxide dismutase activities in the testes, epididymis, and hypothalamus of male Wistar rats treated with mercuric chloride for a period of 28 days. Results are shown as mean ± SD (*n* = 8), *p* < 0.01, 0.0001 (**, ****), ns: not significant. CAT: catalase, SOD: superoxide dismutatse.

**Figure 5:**
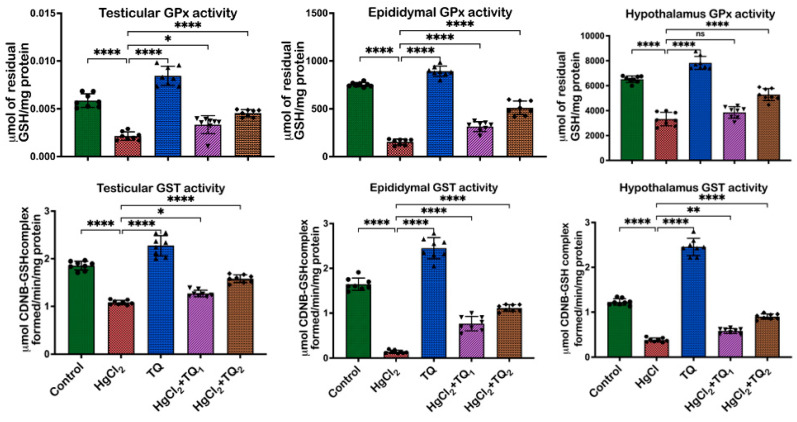
The effect of Thymoquinone (TQ) administration on Glutathione peroxidase (GPx) and Glutathione-S-Transferase (GST) activities in the testes, epididymis, and hypothalamus of experimental rats treated with mercuric chloride for a period of 28 days. Results are shown as mean ± SD (*n* = 8), *p* < 0.05, 0.01, 0.0001 (*, **, ****), ns: not significant.

**Figure 6:**
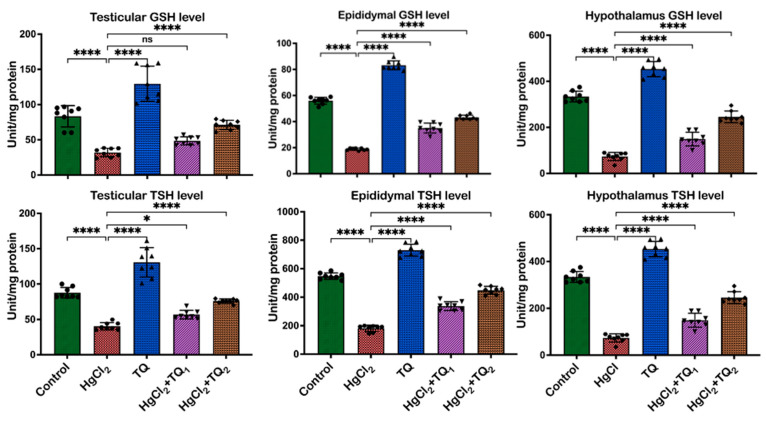
The effect of Thymoquinone (TQ) treatment on the levels of glutathione (GSH) and total sulfhydryl groups (TSH) in the testes, epididymis, and hypothalamus of male Wistar rats administered with mercuric chloride for a period of 28 days. Results are shown as mean ± SD (*n* = 8), *p* < 0.05, 0.0001 (*, ****), ns: not significant.

Figure 4, Figure 5 and Figure 6 depict the effect of TQ co-treatment on the antioxidant status of rats treated with HgCl_2_. In the testes of rats treated with HgCl_2_, a marked decline (*p* < 0.05) was observed in the activities of CAT [F (4, 35) = 289.9, *p* < 0.0001, 52.28%], SOD [F (4, 35) = 137.8, *p* < 0.0001, 57.77%], GST [F (4, 35) = 133.0, *p* < 0.0001, 41.67%], GPx [F (4, 35) = 86.84, *p* < 0.0001, 63.39%], along with the levels of GSH [F (4, 35) = 58.42, *p* < 0.0001, 61.68%] and TSH [F (4, 35) = 85.07, *p* < 0.0001, 54.10%]. Remarkably, co-treatment with TQ raised (*p* < 0.05) these parameters, particularly evident at the higher dose (5.0 mg/kg) of TQ, where SOD, GST, and GPx increased by 108.62%, 45.84%, and 112.34%, respectively, compared to the HgCl_2_ group. Furthermore, in the epididymis, HgCl_2_ treatment substantially reduced the activities of CAT (F (4, 35) = 448.6, *p* < 0.0001, 85.13%), SOD (F (4, 35) = 317.8, *p* < 0.0001, 42.91%), GST (F (4, 35) = 289.5, *p* < 0.0001, 91.50%), GPx (79.56%), along with the levels of GSH (F (4, 35) = 641.8, *p* < 0.0001, 66.43%) and TSH (F (4, 35) = 386.5, *p* < 0.0001, 66.79%). Contrastingly, co-treatment with TQ significantly improved these parameters. Moreover, treatment of experimental animals with HgCl_2_ significantly downgraded the hypothalamic activities of CAT [F (4, 35) = 107.6, *p* < 0.0001, 77.45%], SOD [F (4, 35) = 176.4, *p* < 0.0001, 58.98%], GST [F (4, 35) = 530.4, *p* < 0.0001, 68.05%], and GPx [F (4, 35) = 126.1, *p* < 0.0001, 50.72%]. The endogenous levels of GSH [F (4, 35) = 176.4, *p* < 0.0001] were equally reduced by 84.13% and TSH [F (4, 35) = 261.0, by 77.80%] upon HgCl_2_ exposure in the hypothalamus. However, subsequent co-treatment with TQ ameliorated (*p* < 0.05) these disruptions and restored the antioxidant capacity of the hypothalamus.

### 3.6. TQ Co-Treatment Attenuated HgCl_2_-Induced Oxidative Stress in the Reproductive Tissues of Rats

**Figure 7:**
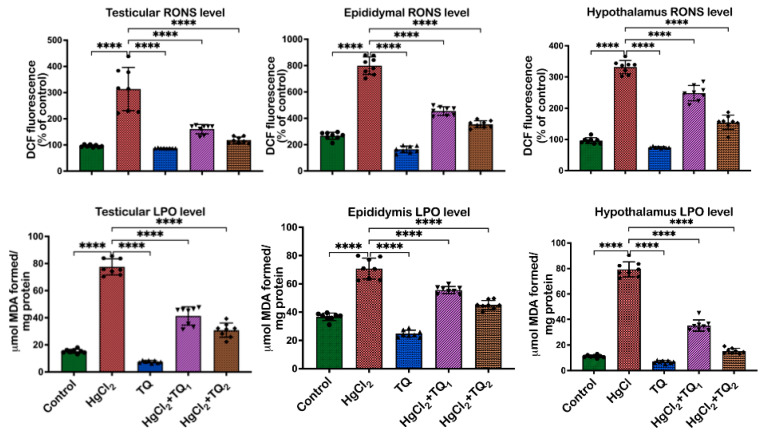
The effect of Thymoquinone (TQ) on reactive oxygen and nitrogen species (RONS) and lipid peroxidation (LPO) in experimental rat epididymis, testes, and hypothalamus following treatment with mercuric chloride for 28 days. Results are shown as mean ± SD (*n* = 8), *p* < 0.0001 (****).

**Figure 8:**
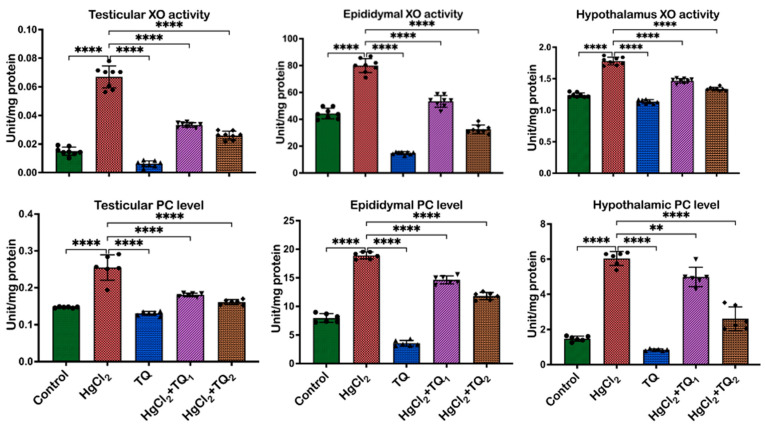
The effect of Thymoquinone on xanthine oxidase (XO; *n* = 8) activity and protein carbonyl (PC; *n* = 6) level in experimental rat epididymis, testes, and hypothalamus co-treated with mercuric chloride for 28 days. Results are shown as mean ± SD, *p* < 0.01, 0.0001 (**, ****).

The ability of TQ co-treatment to attenuate HgCl_2_-provoked oxidative stress in the rats’ reproductive tissues is displayed in Figure 7 and Figure 8. Exposure of rats to HgCl_2_ caused an upsurge (*p* < 0.05) in RONS level [F (4, 35) = 47.19, *p* < 0.0001], LPO level [F (4, 35) = 269.8, *p* < 0.0001] and XO activity [F (4, 35) = 264.5, *p* < 0.0001] by 407.79%, 345.84%, and 225.92% in the testes, relative to the control. However, TQ, alongside HgCl_2_ (2.5 and 5 mg/kg doses), effectively ameliorated these increases. Specifically, 2.5 mg/kg co-treatment reduced RONS and LPO levels and XO activity by 46.71%, 50.12%, and 48.69%, respectively, while treatment with the higher dose of TQ (5 mg/kg) further decreased RONS and LPO level and XO activity, showing reductions by 60.25%, 61.07%, and 62.21%, respectively, in comparison to the HgCl_2_ treatment alone in the testes. In the epididymis, HgCl_2_ exposure increased RONS [F (4, 35) = 314.2, *p* < 0.0001], LPO levels [F (4, 35) = 149.2, *p* < 0.0001], as well as XO [F (4, 35) = 325.3, *p* < 0.0001] activity, by 92.86%, 80.28%, and 198.17%, respectively. Remarkably, the 2.5 mg/kg treatment attenuated (*p* < 0.05) these increases, resulting in decreases of 21.48%, 33.29%, and 24.35%, respectively. The group of rats co-treated with the high dose of TQ (5.0 mg/kg) had these levels reduced by 36.26%, 59.16%, and 55.55%, respectively, compared to the HgCl_2_-only group. Additionally, the hypothalamus of experimental rats demonstrated a marked increase in RONS [F (4, 35) = 273.5, *p* < 0.0001] and LPO levels [F (4, 35) = 587.8, *p* < 0.0001], as well as the activity of XO [F (4, 35) = 312.0, *p* < 0.0001], by 622.94%, 47.49%, and 242.38%, respectively, following HgCl_2_ administration alone. Contrastingly, co-treatment with 2.5 mg/kg of TQ reduced (*p* < 0.05) the observed increments by 55.63%, 20.08%, and 24.35%, respectively. Animals that received the higher dose (5.0 mg/kg) displayed pronounced protective effects, with decreases (*p* < 0.05) in RONS and LPO levels, and XO activity by 80.51%, 26.33%, and 53.08%, respectively, compared to the HgCl_2_-treated group alone. The level of protein carbonyl was raised (*p* < 0.05) in the testes [F (4, 25) = 54.68, *p* < 0.0001, 73.29%], epididymis [F (4, 25) = 505.9, *p* < 0.0001, 137.66%], and hypothalamus [F (4, 25) = 160.2, *p* < 0.0001, 307.63%] upon HgCl_2_ administration alone. However, treatment of rats with TQ (low and high doses) resulted in a significant reduction in the levels of PC in the testes (29.34% and 37.60%), epididymis (22.67% and 37.63%), and hypothalamus (17.38% and 56.68%).

### 3.7. TQ Co-Administration Reversed Inflammation Caused by HgCl_2_ Exposure in the Testes, Epididymis, and Hypothalamus of Rats

**Figure 9:**
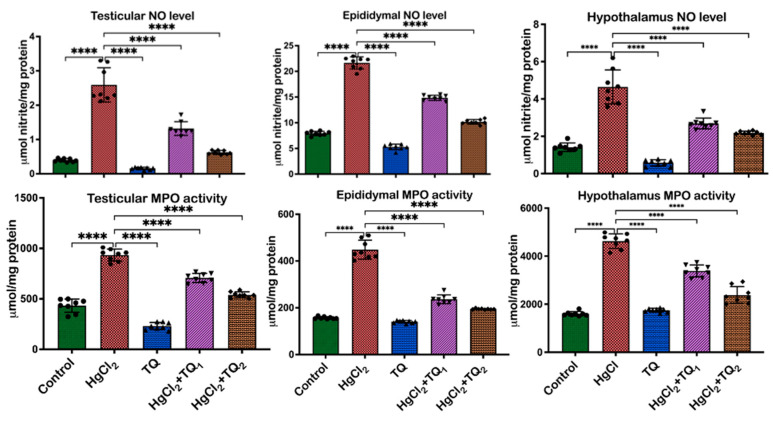
The effect of Thymoquinone (TQ) on inflammatory biomarkers—nitric oxide (NO) and myeloperoxidase (MPO) in the testes, epididymis, and hypothalamus of rats co-treated with mercuric chloride for 28 days. Results are shown as mean ± SD (*n* = 8), *p* < 0.0001 (****).

**Figure 10:**
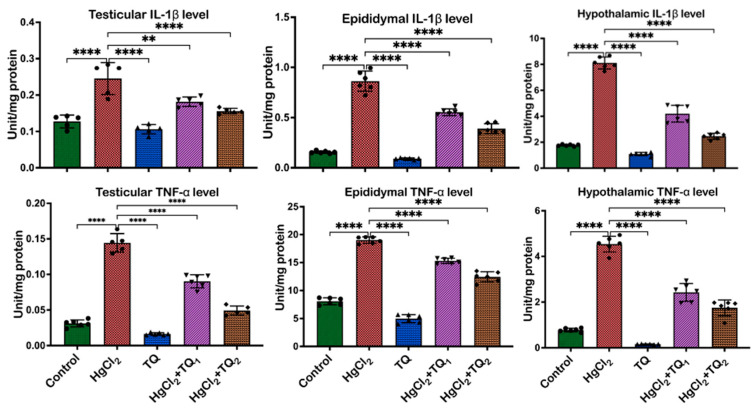
The effect of Thymoquinone (TQ) administration on biomarkers of inflammation—level of Tumour Necrosis Factor alpha (TNF-α) and Interleukin-1 beta (IL-1β) in experimental rat epididymis, testes, and hypothalamus following treatment with mercuric chloride—treated for 28 days. Results are shown as mean ± SD (*n* = 6), *p* < 0.01, 0.0001 (**, ****).

Figure 9 and Figure 10 evidence the anti-inflammatory activity of TQ co-administration in the testes, epididymis, and hypothalamus of rats treated with HgCl_2_. There was an increase (*p* < 0.05) in the levels of NO [F (4, 35) = 130.7, *p* < 0.0001], MPO activity [F (4, 35) = 237.6, *p* < 0.0001], TNF-α level [F (4, 25) = 219.6, F (4, 25) = 216.4], and IL-1β level [F (4, 20) = 27.67, *p* < 0.0001] in the testes of animals by 556.03%, 363.41%, 115.90%, and 92.39%, compared to the control group. Contrastingly, animals co-treated with TQ (2.5 mg/kg dose) effectively reduced these levels: NO by 49.03%, TNF-α by 37.46%, MPO by 24.29%, and IL-1β by 25.89%. Rats in the 5.0 mg/kg co-treatment group exhibited more distinct reductions with decreases of 75.89%, 65.78%, 42.24%, and 36.45%, respectively, compared to animals that received HgCl_2_ alone. The epididymis of rats post-HgCl_2_ exposure showed increases (*p* < 0.05) in NO [F (4, 35) = 721.9, *p* < 0.0001], MPO [F (4, 35) = 301.3, *p* < 0.0001], TNF-α [F (4, 25) = 405.3, *p* < 0.0001], and IL-1β [F (4, 25) = 215.4, *p* < 0.0001] by 173.34%, 135.97%, 185.24%, and 452.82%, respectively, compared to control levels. However, 2.5 mg/kg treatment significantly countered these effects, reducing NO levels by 31.42%, TNF-α by 19.52%, MPO activity by 47.26%, and IL-1β by 35.80%. Administration of TQ at a dose of 5.0 mg/kg reduced (*p* < 0.05) these biomarkers by 53.05%, 34.61%, 56.60%, and 54.70%, respectively, compared to the HgCl_2_-only group in the epididymis. Following HgCl_2_ exposure, the hypothalamus exhibited increases (*p* < 0.05) in the levels of NO [F (4, 35) = 93.99, *p* < 0.0001, 226.62%], TNF-α [F (4, 25) = 219.6, *p* < 0.0001, 481.46%], MPO [F (4, 35) = 217.6, *p* < 0.0001, 190.06%], and IL-1β [F (4, 25) = 337.2, *p* < 0.0001, 359.12%] compared to the control. However, TQ co-treatment, at 2.5 and 5.0 mg/kg, effectively attenuated these escalations, as demonstrated by decreases (*p* < 0.05) of 40.99% and 52.56%, 46.54% and 61.35%, 26.43% and 48.75%, and 48.25% and 69.60%, respectively, in the hypothalamus.

### 3.8. TQ Co-Treatment Assuaged Apoptosis and Cellular Damage in Experimental Rats Treated with HgCl_2_

**Figure 11:**
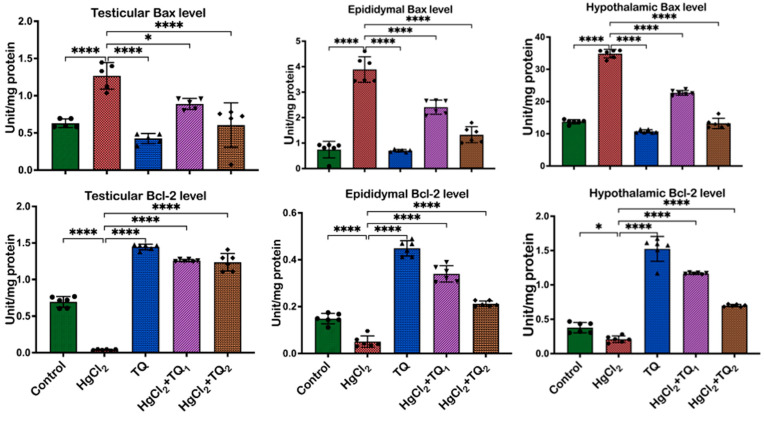
The effect of Thymoquinone (TQ) administration on biomarkers of apoptotic Bcl-2 and Bax in the epididymis, testes, and hypothalamus of male Wistar rats treated with mercuric chloride for a period of 28 days. Results are shown as mean ± SD (*n* = 6), *p* < 0.05, 0.0001 (*, ****).

**Figure 12:**
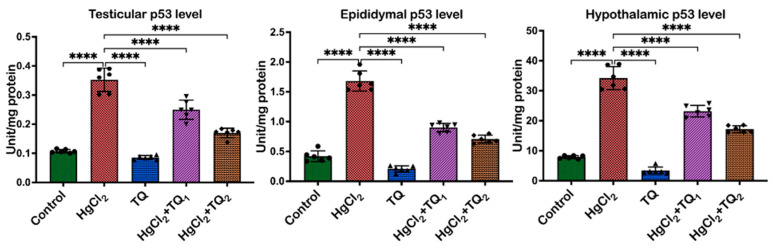
The effect of Thymoquinone (TQ) on the level of TProtein 53 (Tp53), a marker of DNA damage in the testes, epididymis, and hypothalamus of mercuric chloride-treated male Wistar rats for 28 days. Results are shown as mean ± SD (*n* = 6), *p* < 0.0001 (****).

The results of TQ supplementation against HgCl_2_-induced apoptosis are depicted in Figure 11 and Figure 12. In the testis, animals treated with HgCl_2_ only led to a reduction (*p* < 0.05) of Bcl-2 [F (4, 25) = 463.8, *p* < 0.0001] by 94.17%. Conversely, compared to control levels, Bax [F (4, 25) = 27.94, *p* < 0.0001] and P53 [F (4, 25) = 117.7, *p* < 0.0001] levels increased by 100.73% and 228.33%, respectively. However, TQ co-treatment significantly reversed these alterations in Bcl-2 expression. Bax and p53 expressions, on the other hand, decreased by 29.71% and 52.09% in the 2.5 mg/kg treatment, and by 29.12% and 51.77% in the 5.0 mg/kg treatment, respectively, in the testes, when compared to HgCl_2_ treatment alone. In the epididymis, HgCl_2_ treatment markedly decreased the Bcl-2 level [F (4, 25) = 211.9, *p* < 0.0001, 65.87%], whereas increasing the expressions of Bax [F (4, 25) = 101.6, *p* < 0.0001, 422.09%] and P53 [F (4, 25) = 199.1, *p* < 0.0001, 300.29%] relative to the control group. However, Bcl-2 levels rose by 568.64% and 317.60%, while Bax expression decreased by 38.04% and 65.95%, and P53 expression by 46.45% and 57.99% for 2.5 and 5.0 mg/kg doses, respectively, compared to the HgCl_2_-only treatment. Furthermore, Bcl-2 expression [F (4, 25) = 215.9, *p* < 0.0001] decreased by 44.54%, while Bax [F (4, 25) = 540.9, *p* < 0.0001] and P53 protein level [F (4, 25) = 211.6, *p* < 0.0001] increased by 153.82% and 333.57%, respectively, compared to control levels in the hypothalamus. However, low- and high-dose TQ co-treatment amplified Bcl-2 expression by 455.08% and 233.24%, respectively, and reduced Bax and p53 expressions by 34.89% and 61.95%, and 32.22% and 49.71%, respectively.

### 3.9. TQ Reduced HgCl_2_-Induced Testicular and Epididymal Histomorphometry Alteration

**Figure 13:**
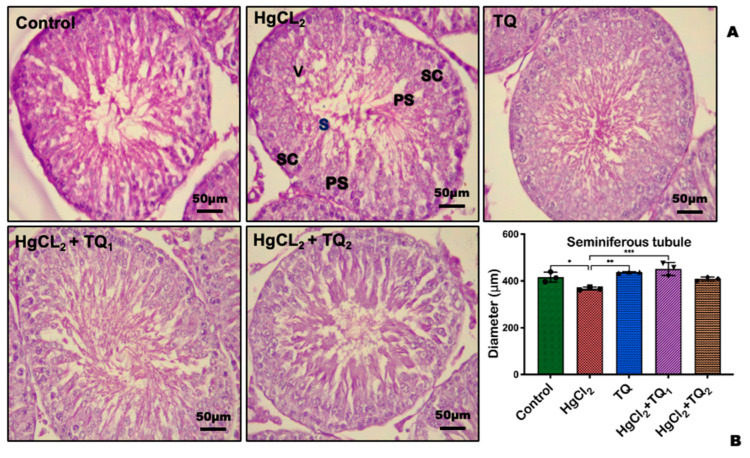
The effect of TQ on testicular histoarchitectural following treatment of experimental rats with HgCl_2_ for 28 consecutive days. The controls and TQ-treated groups exhibit typical testicular structure, characterised by well-organised seminiferous tubules and germinal epithelium, primary spermatocyte, Leydig and Sertoli cells (SC), and spermatids. The HgCl_2_-only exposed rat shows mild testicular vacuolation of the seminiferous tubule, vacuolation of Sertoli cells (V), and fewer spermatids (Ss) and primary spermatocytes (PSs) (**A**). Each bar represents the mean values, with asterisks indicating significant differences at * *p* < 0.05, ** *p* < 0.01, and *** *p* < 0.001 versus control and HgCl_2_, as determined from histomorphometric data (**B**). H & E section of the testis, scale bar = 50 µm, magnification: ×400.

**Figure 14:**
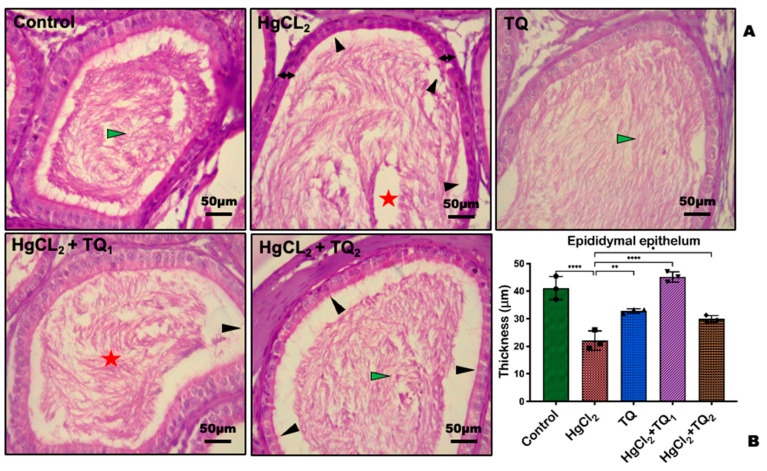
Epididymal section of Thymoquinone (TQ) and mercury chloride (HgCl_2_). The control and TQ-treated groups show typical epididymal structures with well-organised epididymal epithelium and mature stored spermatozoa. The HgCl_2_-only exposed rat exhibits epididymal degeneration, characterised by a reduction in stored mature spermatozoa (**A**). Epididymal epithelium thickness (double arrow), Epididymal lumen with stored mature sperm cells (green arrowhead), Epididymal lumen with few stored disorganised sperm cells (red star), aggregation of sperm cell from the epididymal epithelium (black arrowhead). Histomorphometric data of the epididymis measeured with ImageJ software (version 1.50i) (**B**). Each bar represents mean values with asterisks indicating significant differences at ** *p* < 0.01, and **** *p* < 0.0001 versus control and HgCl_2_. H & E section of the testis, scale bar = 50 µm, magnification: ×400.

Corresponding photomicrographs depict the histological findings (Figure 13A and Figure 14A) and histomorphological measurements (Figure 13B and Figure 14B) obtained from the experimental rats’ epididymis and testis treated with HgCl_2_ and TQ. Figure 13 and Figure 14 show the effect of TQ on the histomorphometry of testicular and epididymal tissues in HgCl_2_-treated rats. HgCl_2_-only exposed rats demonstrated significant atrophy, vacuolation of Sertoli cells, and a notable decrease in the seminiferous tubular diameter [F (4, 10) = 11.30, *p* = 0.001, Figure 13], as well as a reduction in epididymal epithelial thickness [F (4, 10) = 35.61, *p* < 0.0001, Figure 14] compared with the control. Conversely, TQ-treated (2.5 and 5 mg/kg) rats partially restored testis and epididymis histoarchitecture with well-organised germinal cells, spermatids, and stored mature sperm cells.

### 3.10. Antioxidant and Anti-Inflammatory Effects Are Promoted Through the Interaction of TQ and PPAR-α or PPAR δ/β Signalling

**Figure 15:**
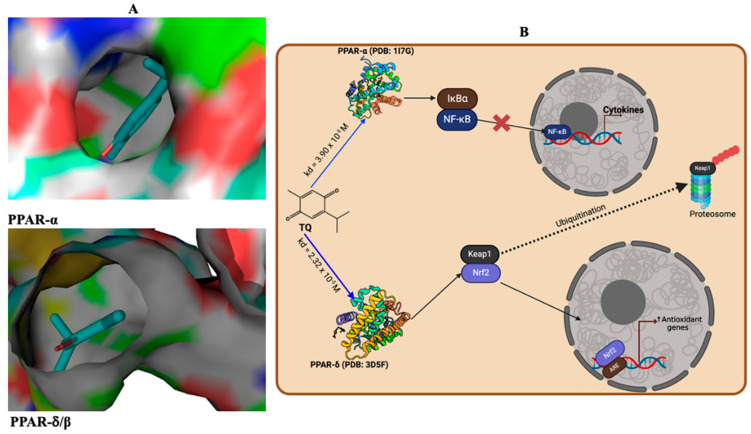
(**A**) Docking analysis illustrating the molecular interaction and the binding of TQ with PPAR-α (PDB: 1I7G) and PPAR-δ/β (PDB: 3D5F) with ΔG values of −5.9 Kcal/mol and −6.2 Kcal/mol. (**B**) TQ can activate PPAR-α or PPAR-δ/β to mediate antioxidant effects or resolve inflammation. Specifically, PPAR-α blocks the triggering of NF-kB, thus preventing the activation of pro-inflammatory factors. Additionally, PPAR-δ/β induces an antioxidant effect by activating Nrf-2, which upregulates the expression of antioxidant genes. TQ: Thymoquinone.

Following the in vivo study results, we conducted docking simulations and analysis to explore TQ’s likelihood to mediate anti-inflammatory and antioxidant effects. The binding constant (Kd) of TQ’s interactions with PPAR-α or PPAR δ/β was assessed. The interaction exhibited a moderate interaction between TQ and PPAR-α or PPAR δ/β, demonstrated by a Kd of 3.90 × 10^5^ M (PPAR-α) and 2.32 × 10^−5^ M (PPAR δ/β) (Figure 15A). Through this interaction, PPAR δ/β can positively engage Nrf-2 to induce antioxidants, while PPAR-α can modulate the function of NF-kB to induce anti-inflammatory responses (Figure 15B). Taken together, these results indicate that TQ might possess antioxidant and anti-inflammatory effects by activating PPAR-α and PPAR δ/β.

## 4. Discussion

Exposure to heavy metals such as mercury may cause abnormalities in the reproductive system, potentially leading to infertility or subfertility in males [40]. This paper aims to investigate the effectiveness of Thymoquinone (TQ), an antioxidant with anti-apoptotic and anti-inflammatory properties, in protecting the hypothalamic-gonadal (HPG) axis of male rats from mercury chloride (HgCl_2_)-induced toxicity.

Organo-somatic index evaluation, amongst other parameters, is vital for assessing toxicity, as changes in these parameters may indicate physiological disturbances in animals [41]. In this study, exposure to HgCl_2_ reduced body weight gain, organ weight, and relative organ weight of the reproductive tissues, as shown in Table 1. The mean body weight for this set of animals has been published in a previous manuscript [13]. A reduction in body weight gain is symptomatic of mercury toxicity, which is often associated with loss of appetite in experimental animals [42]. This observation is in line with other research that reported insignificant changes in body weight gain and organ weights upon exposure to HgCl_2_. However, administration of TQ increased body weight gain and organ weights in the experimental rats, suggesting TQ’s beneficial effect against nutritional derangement response from dietary exposure to HgCl_2_.

Sperm quality, including parameters such as motility, count, viability, and morphology, is a crucial measure of male fertility in toxicological studies [43]. In this study, exposure to HgCl_2_ significantly reduced sperm motility by approximately 27.78% compared to controls, highlighting its adverse effects (Table 2). Sperm motility is essential for fertilisation capabilities, and a decline of 27.78% could jeopardise this function (Table 2). This finding aligns with previous research indicating that HgCl_2_ impairs sperm membrane integrity and induces morphological defects [1].

HgCl_2_ exposure led to a decline in sperm count versus controls, highlighting harmful impacts on the spermatogenesis pathway and consequently on male fertility. A reduced sperm count can lower the chances of successful fertilisation, according to Dabbagh Rezaeiyeh et al. [44], who found similar results where HgCl_2_ disrupted the germinal epithelium, reducing sperm production [2]. Interestingly, semen volume remained consistent across all groups (Table 2), suggesting that while HgCl_2_ affects spermatogenesis, it does not appear to impact seminal fluid production or the volume of ejaculation. This observation contrasts with the results of Altunkaynak et al. [45], who noted a significant decrease in sperm volume when rats were treated with elemental mercury (Hg^0^) compared to HgCl_2_.

While the changes in morphological abnormalities of the sperm between groups post-HgCl_2_ exposure were insignificant, subtle alterations in sperm morphology can affect their functionality [46], as shown in Table 2. The minor increases in abnormalities across the headpiece, midpiece, and tail, albeit statistically insignificant, hint at possible disruptions in spermiogenesis—the final stages of sperm maturation. Previous studies outlined mercury’s ability to induce sperm DNA damage [2,47,48], which might be corroborated by these observed morphological perturbations. Nevertheless, TQ administration was observed to have an insignificant increase in sperm morphological and functional parameters in this study (Table 2). This result contradicts prior findings, which showed that TQ exhibited commendable recuperative actions against various sperm parameter toxicities in rats [45]. The subtle increases in morphological abnormalities even post-TQ treatment, especially in the 5.0 mg/kg co-treatment group, might suggest that TQ’s protective mechanisms do not entirely circumvent the structural disruptions instigated by mercury.

A complex network of hormones regulates the male reproductive system, including LH, FSH, testosterone, and prolactin [49]. These hormones are produced in the testes and pituitary glands and are responsible for various functions such as sperm production, sex drive, and secondary sexual characteristics [50]. LH and FSH stimulate the production of testosterone, which is essential for the development and upkeep of male reproductive and accessory reproductive organs [50]. Prolactin, on the other hand, plays a role in suppressing the production of LH and FSH, which can lead to a decrease in testosterone levels [50]. The significant decreases in testosterone, LH, and FSH in the experimental animal serum upon exposure to HgCl_2_ corroborate the reports delineated by earlier investigations, where HgCl_2_’s toxicological profile encompassed reproductive harm [6,51], as seen in Figure 2. A decline in LH and FSH levels, the primary regulators of spermatogenesis and testosterone synthesis, suggests potential disruptions in the HPG axis (Figure 2). A concomitant reduction in testosterone, a pivotal steroid for maintaining male secondary sexual characteristics, sperm production, and libido, provides evidence of this compromised HPG axis, potentially underscoring reduced Leydig cell functionality or disrupted feedback mechanisms. Conversely, the surge in prolactin levels post-HgCl_2_ exposure presents an intriguing conundrum. In a male physiological context, elevated prolactin can suppress LH and FSH secretion, potentially contributing to the observed declines in these hormones and, by extension, testosterone [52].

In contrast, administering TQ (2.5 and 5.0 mg/kg) conferred therapeutic effects by mitigating the hormonal aberrations induced by HgCl_2_. This normalisation of hormonal levels, particularly testosterone restoration and prolactin reduction, underscores TQ’s potential in safeguarding the endocrine system of male rats (Figure 2). These findings are consistent with previous research, which has shown that TQ can counteract heavy metal-induced endocrine and reproductive derangements [12]. Furthermore, the dose-dependent effectiveness of TQ is evident, as the elevated dose (5.0 mg/kg) results in more pronounced rectifications (515.08%), underscoring its potential therapeutic range. This finding supports the hypothesis that TQ may have an influence at various levels—shielding testicular Leydig cells, revitalising the HPG axis, and theoretically directly modulating steroidogenic enzymes.

Mercury’s disruptive influence on the reproductive system, particularly on the testes, has been a focal point of numerous scientific inquiries in recent years [40]. Due to its pivotal role in spermatogenesis, the testicular tissue is a highly metabolically active site, relying on specialised enzymes to maintain its structure and function. Alkaline phosphatase (ALP) plays a crucial role in phosphate metabolism and membrane transport, supporting the development and maturation of germ cells within the seminiferous epithelium [53]. Acid phosphatase (ACP), primarily a lysosomal enzyme, is involved in sperm maturation. At the same time, lactate dehydrogenase (LDH) is essential for energy metabolism in spermatogenic cells, supporting sperm motility and viability through anaerobic glycolysis [54]. G-6-PD contributes to redox homeostasis by generating NADPH, which protects testicular cells from oxidative damage and supports steroidogenic activity in Leydig cells [55]. The current study observed a significant decrease in the following enzymes: ALP, G-6-PD, LDH, and ACP. These activities are relevant for optimal testicular function upon exposure to HgCl_2_ (Figure 3). Reductions in ALP and ACP activities, which are closely correlated with spermatogenic activity and testicular cellular function, suggest that exposure to HgCl_2_ may adversely affect sperm maturation processes (Figure 3). Similarly, the downturn in LDH, a crucial enzyme for cellular energetics, and G-6-PD, central to redox balance, alludes to a dual blow: an energetic crisis and heightened vulnerability to oxidative perturbations. This complements the findings of Jahan, Azad [2], where mercury exposure resulted in compromised sperm production and increased oxidative damage. Conversely, TQ, especially at the higher dose (5.0 mg/kg), alleviated the HgCl_2_-induced reductions in enzyme activity and augmented them to levels surpassing those seen in controls (Figure 3). This resonates with the earlier findings about TQ’s protective role against drug and heavy metal-induced reproductive damage [10,12]. Testicular cells, particularly spermatogenic and Leydig cells, are susceptible to oxidative stress, which can impair enzyme function and reduce fertility [56]. TQ scavenges ROS and upregulates endogenous antioxidant defence systems, preserving the structural integrity and function of testicular tissues. By mitigating lipid peroxidation and oxidative damage in the testes, TQ helps restore or enhance the activity of these testicular enzymes.

A consistent theme across studies on mercury toxicity is the perturbation of oxidative balance [2,6,18]. In this study, a significant reduction in some enzymatic antioxidant enzymes—CAT, GST, SOD, and GPx activities—was observed (Figure 4 and Figure 5), reflecting the adverse impact of HgCl_2_ on the enzymatic antioxidants of the male rats’ reproductive tissues. The disruption of these enzymes signifies an overwhelmed oxidative defence system, rendering the cells vulnerable to oxidative damage. This aligns with the established understanding that HgCl_2_ stimulates the production of ROS, which, in turn, can cause cellular damage, inflammation, and apoptosis, central to mercury’s reproductive toxicity [4,8]. Furthermore, the reduction in glutathione (GSH) and total sulfhydryl (TSH) levels indicates depleted cellular thiols Figure 6. GSH, a tripeptide, is fundamental in cellular defence against oxidative stress, and its depletion is a well-recognised marker of cellular oxidative damage [57]. However, findings from this study demonstrate the protective effects of TQ as co-treatment with TQ, specifically at higher doses (5 mg/kg), significantly increased antioxidant enzymes and accessory biomolecules initially depleted by HgCl_2_ (Figure 6). This result suggests that TQ might be acting by scavenging the ROS produced, upregulating the antioxidant defence genes, or both. The notable increase in GST activity, especially in the epididymis and hypothalamus following TQ co-treatment, emphasises its role in detoxification. GST is known to conjugate GSH to various electrophilic compounds, aiding their removal and neutralisation [58]. Therefore, the increased activity indicates a mechanism where TQ potentially induces GST expression or provides the necessary cofactors to optimise its activity.

Moreover, the capacity of HgCl_2_ to induce oxidative stress is demonstrated by notable elevations in oxidative damage biomarkers—including RONS, LPO, protein carbonyl content, and XO activity—across the testes, epididymis, and hypothalamus of exposed rats [53], as illustrated in Figure 7 and Figure 8. These findings are consistent with previously established data, which show that mercury exposure is associated with an imbalance in the oxidative balance of reproductive tissues in rats [2,6,18]. The rise in LPO levels, a marker of cellular lipid membrane damage, underscores the direct damage inflicted on cellular structures by ROS. Elevated levels of LPO are indicative of compromised membrane integrity and function, potentially leading to cellular dysfunction and death [59]. Similarly, the surge in XO enzyme activity, involved in the synthesis of xanthine via oxidation of hypoxanthine, additionally contributes to ROS generation and consequently intensifies oxidative stress [60].

Additionally, the increase in RONS further corroborates the notion of heightened oxidative and nitrosative stress upon HgCl_2_ exposure. Contrastingly, TQ significantly protected the examined tissues from HgCl_2_-induced oxidative damage (Figure 7 and Figure 8). The protective effects of TQ, particularly at higher doses, resonate with its previously documented antioxidant [15,61]. It appears that TQ either directly scavenges the free radicals generated by HgCl_2_ exposure or upregulates the cellular antioxidant defence system, thereby restoring the oxidative balance. The significant reduction in LPO, XO, and RONS in the testes upon TQ co-treatment suggests an amelioration of HgCl_2_-induced cellular and enzymatic damage. This has profound implications for male fertility, as the testes are pivotal for sperm production, the epididymis for sperm maturation and storage, and the hypothalamus for regulating numerous physiological functions, including the synthesis of reproductive hormones. Oxidative stress in these organs can compromise spermatogenesis, resulting in reduced sperm count, decreased motility, and increased sperm abnormalities [15,61]. However, the attenuation of oxidative markers in the epididymis suggests that TQ may preserve the structural and functional integrity of maturing sperm, ensuring their viability and motility, and maintaining endocrine homeostasis under oxidative insults.

It is evident from previous work that damaged cellular components, resulting from oxidative stress, can act as danger signals or “alarmins” that are recognised by specific receptors on immune cells, leading to the activation of various inflammatory pathways [62]. Furthermore, products of lipid peroxidation are capable of activating the leucine-rich repeat (LRR), nucleotide-binding oligomerisation domain-like receptors (NODs), and pyrin domain-containing protein 3 (NLRP3) inflammasome. This activation subsequently promotes the production and secretion of pro-inflammatory cytokines, including TNF-α and IL-1β [59]. Moreover, ROS can directly activate transcription factors such as NF-κB, which plays a central role in the expression of genes responsible for inflammatory responses [63]. Therefore, the sustained oxidative stress, potentially caused by HgCl_2_ exposure, can perpetuate a chronic inflammatory state in the testes, epididymis, and hypothalamus. Inflammation, depending on the duration, is a two-edged sword. While acute inflammation is protective and aids tissue repair, chronic or persistent inflammation is detrimental and has been implicated in many pathological conditions, including reproductive dysfunctions [64]. In this study, a pronounced inflammatory response was observed in the examined organs upon HgCl_2_ administration, as evidenced by the elevation in inflammatory markers, NO, MPO, IL-1β, and TNF-α (Figure 9 and Figure 10). Although a crucial signalling molecule in numerous physiological processes, NO can react with superoxide radicals at elevated levels to form peroxynitrite, a potent oxidant that can induce cellular damage [65]. The significant upsurge in NO post-HgCl_2_ exposure indicates the oxidative and nitrative stress inflicted on the examined tissues. The concomitant rise in pro-inflammatory cytokines observed in the current study—TNF-α and IL-1β—and the myeloperoxidase marker of neutrophil infiltration, suggests an active inflammatory process (Figure 9 and Figure 10).

The increase in protein carbonyl levels further highlights oxidative protein damage, potentially compromising the structural and functional integrity of proteins within the testes. Additionally, the significant rise in IL-1β levels, a potent pro-inflammatory cytokine, coupled with the elevation in other inflammatory markers (NO and MPO), suggests an epididymal environment that may not be conducive to sperm maturation and might compromise sperm motility and function. Moreover, chronic inflammation in the hypothalamus can potentially disrupt hormonal homeostasis, affecting downstream reproductive processes, such as hormone production, and other vital functions regulated by this brain region [66]. This inflammatory and oxidative milieu can impair spermatogenesis, reduce testosterone synthesis, and affect overall testicular health. On the other hand, the administration of TQ effectively counteracted the HgCl_2_-induced inflammation in the testes, epididymis, and hypothalamus of rats. The higher dose (5.0 mg/kg) manifested more pronounced effects, suggesting a dose-dependent protective role of TQ. TQ has been shown to exert an anti-inflammatory effect by inhibiting inflammatory cytokines and processes, such as TNF-α, inducible NOS, cyclooxygenase-2 (COX-2), 5-lipoxygenase, and cyclin D1 [67]. TQ also modulates the nuclear factor-κΒ and mitogen-activated protein kinase signalling pathways, which regulate inflammation and cell proliferation [68].

Apoptosis is an essential biological mechanism that ensures cellular balance by removing impaired or redundant cells; the balance between pro- and anti-apoptotic biomolecules determines cellular fate [69]. Bcl-2-associated X protein-Bax induces mitochondrial outer membrane permeabilisation, thereby promoting the release of cytochrome c to the cytosol [70]. Once released, cytochrome c triggers a torrent of events that culminate in cell death, rendering Bax a pro-apoptotic protein. Bax forms pores or channels in the mitochondrial membrane, which facilitates further discharge of cytochrome c and additional pro-apoptotic factors [70]. On the other hand, Bcl-2 (B-cell lymphoma 2) prevents mitochondrial membrane permeabilisation and consequent discharge of cytochrome c by forming heterodimers with Bax, thereby inhibiting cell death [71]. TP53 is a protein-suppressing tumour development and also regulates cellular stress responses, including DNA damage [72]. It is a transcription factor that activates genes involved in cell cycle arrest, DNA repair, senescence, and apoptosis. This transcription factor promotes cell death by upregulating the expression of pro-apoptotic genes, including Bax, while simultaneously repressing anti-apoptotic genes, such as Bcl-2 [73]. By modulating the balance between pro- and anti-apoptotic factors, p53 helps to eliminate cells with irreparable DNA damage or those at risk of becoming cancerous, thus earning it the title “guardian of the genome [72].”

Additionally, p53 can directly induce apoptosis through transcription-independent mechanisms, such as interacting with proteins favouring apoptosis or those stimulating the permeabilisation of mitochondrial outer membrane [73]. The significant decrease in Bcl-2 expression observed in the HgCl_2_-alone group across the testes, epididymis, and hypothalamus indicates the potent ability to induce apoptosis (Figure 11 and Figure 12). Since Bcl-2 is vital in inhibiting apoptosis, its reduction would predispose cells to death, possibly accounting for the observed pathological changes and functional impairments in the reproductive tissues and the regulatory centre (hypothalamus). Concomitantly, the substantial upregulation of Bax and P53 upon HgCl_2_ exposure underscores apoptotic pathway activation. Bax initiates the intrinsic apoptotic cascade by promoting cytochrome c release from the mitochondria, while P53 facilitates apoptosis in response to DNA damage [72]. The protective role of TQ was evident, as shown by the significant increase in Bcl-2 expression across all tissues upon TQ co-administration, implying that TQ can bolster cellular defences against apoptosis (Figure 11 and Figure 12), thereby promoting cell survival. This is particularly crucial in the testes, where preserving the population of various cells, including the sperm-producing germ cells and testosterone-producing Leydig cells, is imperative for maintaining reproductive health. In the epididymis, safeguarding cells ensure optimal conditions for sperm maturation, and in the hypothalamus, neuronal survival is essential for endocrine and physiological regulation [74,75].

Additionally, the concurrent reduction in Bax and P53 expressions upon TQ treatment in all tissues suggests that TQ can block the apoptotic signals induced by HgCl_2_. This observation concurs with earlier studies [76,77]. The significant downregulation of these pro-apoptotic molecules suggests that TQ may prevent the cellular cascades that lead to apoptosis, thereby safeguarding cellular integrity and function.

Our histomorphometry findings are consistent with our results on sperm morphology, although with limited testicular tubules, histopathological derangement of the germ cell layer, and Sertoli cells. Profound histopathological derangement might adversely impair spermatogenesis. Considerable detachment of spermatozoa from the germinal epithelium of the epididymis was observed, particularly in the group treated with HgCl_2_ alone, which might adversely impact the sperm maturation process, count, viability, and motility (Table 2) following oral exposure to HgCl_2_. This finding confirmed earlier studies that indicated ultrastructural impairment of the testis and epididymis in experimental animals concerning the exposure to HgCl_2_ [78,79]. TQ-treated rat cohorts showed improved histoarchitecture and slightly improved interactions with the epididymal epithelium, which was not the case at higher doses of TQ.

PPARs—Peroxisome proliferator-activated receptors—particularly PPAR-γ, PPAR-α, and PPAR δ/β, are an excellent therapeutic target. They have been known to induce antioxidant and anti-inflammatory functions [80]. They promote optimal health by conferring antioxidant and anti-inflammatory responses [81,82,83,84]. Molecular docking simulations demonstrated that PPAR δ/β positively engage Nrf-2 and modulate NF-kB function, promoting antioxidants and anti-inflammatory responses. Thus, TQ anti-oxidative and anti-inflammatory effects are by agonistic activation of PPAR-α and PPAR δ/β. Our in silico studies showed that TQ could act as a PPAR-α and PPAR δ/β agonist, thereby increasing its potential to activate Nrf-2 and inhibit NF-kB. The activation of Nrf-2 upregulates the expression of cytoprotective genes, while inhibition of Nf-kB represses the transcription/translation of pro-inflammatory mediators [80]. Upon confirming our current observations in conjunction with previous studies, we propose that TQ mitigates the reproductive toxicities induced by HgCl_2_ by increasing endogenous sources of antioxidant enzymes, which can counteract the generation of ROS initiated by HgCl_2_. These mechanisms diminish pro-inflammatory mediators, apoptosis, and the histological aberrations orchestrated by the toxicant to the reproductive system. Specifically, SOD catalyses the conversion of superoxide anion radicals resulting from HgCl_2_-induced oxidative damage to DNA—arising from the excessive release of nucleotide (purines) and their precursor/breakdown (hypoxanthine, and xanthine) components—into peroxide of hydrogen. In the absence of transformation into water by CAT or GPx, the resultant hydrogen peroxide has the potential to react with labile iron species (Fe^2+^/Fe^3+^) in the Fenton reaction, generating hydroxyl radicals, which represent a more toxic form of ROS. These hydroxyl radicals may interact with membrane lipids, culminating in lipid peroxidation, as evidenced by increased levels of MDA as a measure of LPO.

**Figure 16:**
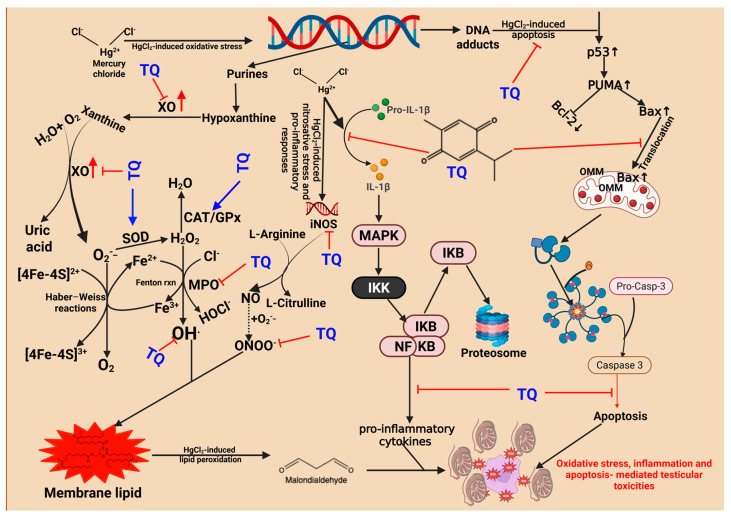
Proposed mechanism of action indicating how Thymoquinone (TQ) suppresses mercuric chloride alteration of male Wistar rats’ testes, epididymis, and hypothalamus after a 28-day study. The activation of mitogen-activated protein kinase (MAPK), nuclear factor erythroid 2-related factor 2 (Nrf2), and the formation of DNA adducts are integral components of the cellular responses to stress and damage caused by mercury exposure. When activated, NF-κB mediates the transcription of pro-inflammatory genes, resulting in inflammation. DNA adducts increase the expression of p53, which subsequently induces PUMA expression. PUMA activates Bax, leading Bax to enter the mitochondria and trigger MPTP, releasing cytochrome c. Cytochrome c combines with Apaf 1 and procaspase 9 to form the apoptosome, which converts procaspase-9 into active Caspase 9. Caspase-9 then activates procaspase 3 to form active caspase-3, culminating in programmed cell death. Thymoquinone inhibits both inflammation and DNA adduct formation. It also enhances the expression of detoxification enzymes, including heme oxygenase 1 (HO 1) and glutathione -s-transferase (GST), as well as other antioxidant enzymes via Nrf2 activation. Created in Biorender. Uche Arunsi. (2025) https://BioRender.com.

Additionally, if hydrogen peroxide is not efficiently removed, it can react with tissue chlorides in the presence of myeloperoxidase (MPO), resulting in the formation of hypochlorous acid, a reactive oxygen species that participates in pro-inflammatory processes within tissues. TQ has been demonstrated to reduce oxidative stress and inflammation occasioned from exposure and subsequent toxicity from mercury by lowering nitric oxide levels linked to increased inducible nitric oxide synthase activity. It also decreases apoptosis in the reproductive system by regulating p53, Bax, and enhancing Bcl-2, ultimately mitigating effects on caspases-9 and -3. These actions help protect the experimental animals from HgCl_2_-induced reproductive toxicity (Figure 16).

This study has some limitations. We did not measure daily food intake, which limits our understanding of how Thymoquinone (TQ) mitigates the effects of mercuric chloride on body weight gain and organ weight. Additionally, the bioavailability of TQ was not assessed, as it was beyond the scope of this study, leaving uncertainty about its systemic absorption and distribution. While molecular docking simulations were employed to identify potential therapeutic targets underlying TQ’s antioxidant and anti-inflammatory effects, these findings are preliminary and require further experimental validation.

## 5. Conclusions

This study found that TQ supplementation reduced HgCl_2_’s negative impact on sperm quality, testicular function, and oxidative balance. Higher TQ doses led to greater improvements in male fertility and mitigated inflammation and cell death in reproductive tissues. These results suggest TQ works via anti-oxidative, anti-inflammatory, and anti-apoptotic mechanisms, making it a promising option for counteracting heavy metal toxicity [13].

## Figures and Tables

**Table 1:** Changes in body weight, as well as relative weights of the testis, epididymides, and hypothalamus, in rats following 28 days of exposure to TQ and HgCl_2_.

	Control	HgCl_2_	TQ	HgCl_2_ + TQ1	HgCl_2_ + TQ2
* Total Rats Per Grouping	(8)	(8)	(8)	(8)	(8)
Initial body weight (g)	162.20 ± 10.72	156.70 ± 7.45	166.70 ± 15.26	162.00 ± 9.62	161.60 ± 13.99
Final body weight (g)	219.00 ± 8.08	209.40 ± 9.64 *	225.10 ± 22.78	227.10 ± 14.60	238.40 ± 13.10 ^#^
Body weight gain (g)	60.86 ± 8.07	57.00 ± 12.45	61.29 ± 14.20 ^ns^	65.25 ± 10.26 ^#,^*	72.63 ± 16.18 ^#,^*
Testes weight (g)	2.50 ± 0.12	2.32 ± 0.07	2.52 ± 0.13 ^ns^	2.63 ± 0.17 ^ns^	2.73 ± 0.22 ^ns^
Relative testes weight (%)	1.14 ± 0.18	1.11 ± 0.08	1.12 ± 0.13 ^ns^	1.16 ± 0.09 ^ns^	1.15 ± 0.10 ^ns^
Epididymides weight (g)	0.29 ± 0.01	0.29 ± 0.01	0.35 ± 0.04 ^ns^	0.30 ± 0.03 ^ns^	0.34 ± 0.05 ^ns^
Relative Epididymides weight (%)	0.13 ± 0.01	0.14 ± 0.01	0.16 ± 0.02 ^ns^	0.13 ± 0.01 ^ns^	0.14 ± 0.01 ^ns^
Hypothalamus Weight (g)	0.09 ± 0.01	0.07 ± 0.03	0.05 ± 0.01 ^ns^	0.08 ± 0.02 ^ns^	0.07 ± 0.02 ^ns^
Relative Hypothalamus Weight (%)	0.04 ± 0.01	0.03 ± 0.01	0.02 ± 0.01 ^ns^	0.04 ± 0.01 ^ns^	0.03 ± 0.01 ^ns^

HgCl_2_ (20 µg/L dissolved in drinking water); TQ1 and TQ2 (2.5 and 5.0 mg/Kg, respectively) body weight; *n* = 8. Data are expressed as mean ± SD. The value of testes and epididymides represents paired organ weights (both testes and both epididymides together) and is calculated as organ weight divided by the corresponding final body weight × 100. Statistical comparisons were performed as follows: the HgCl_2_ group was compared directly with the control group, while the co-treatment groups (HgCl_2_ + TQ1 and HgCl_2_ + TQ2) were compared with the HgCl_2_-only group. * *p* < 0.05 versus control; ^#^ *p* < 0.05 versus HgCl_2_ alone; ns: not significant. HgCl_2_: Mercuric chloride; TQ: Thymoquinone. The initial and final body weight for this set of animals has been published in a previous manuscript [13].

**Table 2:** Sperm analysis and sperm abnormalities of rats following exposure to HgCl_2_ for 28 days.

	Control	HgCl_2_	TQ	HgCl_2_ + TQ1	HgCl_2_ + TQ2
* Total Rats Per Grouping	(8)	(8)	(8)	(8)	(8)
Sperm Functional Analysis					
Motility	90.00 ± 4.62	75.00 ± 5.34 ****	72.50 ± 4.62	67.50 ± 4.62 *	65.00 ± 5.34 **
Viability	96.50 ± 1.60	96.13 ± 1.55	96.50 ± 1.60	96.50 ± 1.64	94.88 ± 4.25
Sperm Volume	5.16 ± 0.05	5.17 ± 0.04	5.17 ± 0.04	5.18 ± 0.03	5.18 ± 0.05
Epididymal Sperm Count	132.40 ± 9.89	117.00 ± 11.10 *	113.00 ± 8.55	101.90 ± 8.25 *	98.50 ± 10.61 **
Sperm Abnormalities					
Abnormality of the Head (%)	2.08 ± 0.27	2.14 ± 0.13	2.15 ± 0.30	2.23 ± 0.31	2.17 ± 0.53
Abnormality of the Midpiece (%)	4.21 ± 0.38	4.65 ± 0.25	4.67 ± 0.35	4.86 ± 0.38	5.14 ± 0.25
Abnormality of the Tail (%)	5.23 ± 0.45	5.61 ± 0.53	5.56 ± 0.56	5.88 ± 0.93	6.25 ± 0.48
Total Abnormality (%)	11.52 ± 0.44	12.41 ± 0.63	12.38 ± 0.63	12.98 ± 1.28	13.56 ± 1.10

HgCl_2_ (20 µg/L dissolved in drinking water); TQ1 and TQ2 (2.5 and 5.0 mg/Kg, respectively) body weight; *n* = 8. Data are expressed as mean ± SD. Statistical comparisons were performed as follows: the HgCl_2_ group was compared directly with the control group, while the co-treatment groups (HgCl_2_ + TQ1 and HgCl_2_ + TQ2) were compared with the HgCl_2_-only group. * *p* < 0.05, ** *p* < 0.01 and **** *p* < 0.0001. HgCl_2_: Mercuric chloride; TQ: Thymoquinone.

## Data Availability

The datasets used and analysed during the current study are available from the corresponding author upon reasonable request.

## Supplementary Materials

The following supporting information can be downloaded at: https://www.mdpi.com/article/10.3390/toxics13100896/s1, Table S1: List of the reagents, kits and chemicals utilised for this study, and S2: Additional information on experimental methodology.
