# Supplementary material for: Thymoquinone Protective Effect Against Mercury-Induced Reproductive Derangement in Rats: In Vivo and In Silico Investigation
Source: Toxics. 2025 Oct 19;13(10):896. doi: 10.3390/toxics13100896 (PMC12567884; doi:10.3390/toxics13100896)
Supplement: Supplementary file 1 [file toxics-13-00896-s001.zip › toxics-3838043-supplementary.pdf]

Supplementary Material (S1)

Table S1: List of the reagents, kits and chemicals utilised for this study

Chemical name	Catalogue No.	Company
**2’,7’-dichlorodihydrofluorescin diacetate**	4091-99-0	Sigma-Aldrich Inc. (St Louis, MO, USA)
**5,5’-dithiobis-(2-nitrobenzoic acid)**	69-78-3	Sigma-Aldrich Inc. (St Louis, MO, USA)
**Copper sulfate pentahydrate**	7758-99-8	Sigma-Aldrich Inc. (St Louis, MO, USA)
**Dipotassium hydrogen phosphate trihydrate**	7758-11-4	AK Scientific, (Union City, CA, USA)
**Epinephrine**	51-43-4	Sigma-Aldrich Inc. (St Louis, MO, USA)
**Folin-Ciocalteu reagent**	125629	J.T Baker (Phillipsburg, PA, USA)
**Glucose 6-phosphate dehydrogenase (G6PD)**	9001-40-5	Elabscience Biotechnology Company (Wuhan, China)
**Hydrogen peroxide (H_2O_2)**	7722-84-1	Sigma-Aldrich Inc. (St Louis, MO, USA)
**Hydrochloric acid**	7647-01-0	Sigma-Aldrich Inc. (St Louis, MO, USA)
**Mercury chloride**		AK Scientific, (Union City, CA, USA)
**O-Dianisidine**	119-90-4	Sigma-Aldrich Inc. (St Louis, MO, USA)
**Potassium Chloride**	7447-40-7	AK Scientific, (Union City, CA, USA)
**Potassium dihydrogen phosphate**	7778-77-0	AK Scientific, (Union City, CA, USA)
**Reduced glutathione (GSH)**	70-18-8	Sigma-Aldrich Inc. (St Louis, MO, USA)
**Sodium azide**	26628-22-8	Sigma-Aldrich Inc. (St Louis, MO, USA)
**Sodium hydroxide**	1310-73-2	Sigma-Aldrich Inc. (St Louis, MO, USA)
**Sodium-Potassium tartrate**	6381-59-5	Sigma-Aldrich Inc. (St Louis, MO, USA)
**Sulphosalicylic acid**	5965-83-3	Sigma-Aldrich Inc. (St Louis, MO, USA)
**Trichloroacetic acid**	76-03-9	Sigma-Aldrich Inc. (St Louis, MO, USA)

**Thiobarbituric acid (TBA)**	504-17-6	Sigma-Aldrich Inc. (St Louis, MO, USA)
**Thymoquinone**	490-91-5	Merck KGaA, Darmstadt, Germany.
**Trichloroacetic acid**	76-03-9	Molychem, (Mumbai India)
**Xanthine**	69-89-6	Sigma-Aldrich Inc. (St Louis, MO, USA)

Supplementary Material S2. Additional information on experimental methodology.

S2.1 Study Conclusion and Euthanasia

After the last treatment with TQ and HgCl2, the rats were weighed, and their final body weights recorded. Twenty-four hours later, blood samples were obtained from the left venous plexus using the retro-orbital method into plain tubes before euthanasia through cervical dislocation after carbon dioxide anesthesia during tissue harvest see Supplementary Materials 1 (SM1) for details. The animals were carefully restrained, and a sterile microcapillary tube was gently inserted into the retro-orbital venous plexus behind the eye. Blood was cautiously aspirated into labelled plain sample bottles. After removing the microcapillary tube, the collected blood was allowed to clot. Preparation of the collected blood serum samples was by centrifugation (condition: 3000 g; 10 minutes) of the clotted blood. The serum was subsequently, labelled, properly (−20 °C) stored until they were required for Enzyme-Linked Immunosorbent hormone assays using ELISA diagnostic kits (Amersham, UK). The hypothalamus, testes, and epididymis were promptly removed, weighed, and subsequently subjected to biochemical and histological examination. The organo-body weight indicators (OBWI) for the epididymis, hypo-thalamus, and testes were appraised by means of the formula OBWI = 100 × gm organ weight /gm body weight. The tissues of interest were harvested and sluiced in sodium phosphate buffer (pH 7.2, 4℃). The samples were stored at −80°C after washing until required for biochemical analysis. The tissues were later homogenised using a Teflon homogeniser (Heidolph Silent Crusher M) in cold potassium phosphate buffer (0.1M, pH 7.4). The protein concentration in the supernatant, obtained following centrifugation of the homogenate, was measured using the Lowry et al., method [19], with bovine serum albumin (BSA) serving as the standard.

S 2.2. Evaluation of Testes, Epididymides and Hypothalamus Antioxidant Biomarkers Status

Testes, epididymides, and hypothalamus samples from experimental rats were homogenised in phosphate buffer (0.05 M; pH 7.4). The tissue homogenates were cen-trifuged (12,000 × g; 15 min; 4°C) to obtain a clear supernatant, which was collected into labelled vials for assessment of

oxidative stress, inflammation, and apoptosis. Superoxide dismutase (SOD) activity was evaluated according to the procedure described by Misra and Fridovich [28], while catalase (CAT) enzyme activity was assessed via Clairborne's method [29]. Enzyme activities for glutathione-S-transferase (GST) and glutathione pe-roxidase (GPx) were measured following the protocols established by Habig [28] and Rotruck [25], respectively. Reduced glutathione (GSH) concentrations were quantified according to the methodology established by Beutler [30], while total sulfhydryl groups (TSH) were assessed utilizing the protocol described by Jollow et al.,[31]. Furthermore, key biomarkers indicative of cellular responses to oxidative stress—namely NRF-2, HO-1, as well as TRX concentrations and TRX-R enzymatic activity—in the testes, epididymis, and hypothalamus of the experimental animals, were measured using rat-specific en-zyme-linked immunosorbent assay (ELISA) kits, adhering to the manufacturer's in-structions as outlined in previous reports[32, 33].

S 2.3. Assessment of RONS and LPO concentrations and XO activity in the testes, epididymides, and hypothalamus of rats

A method utilising the RONS-dependent oxidation of DCFH-DA- 2',7'-dichlorodihydrofluorescein diacetate- to DCF- dichlorofluorescein; was employed to evaluate RONS production in the testes, epididymis, and hypothalamus [33]. Lipid pe-roxidation was measured by assessing the formation of TBARS- thiobarbituric ac-id-reactive substances- in the test sample, following the procedure described by Okhawa [34]. Malondialdehyde, produced by fatty acid peroxidation, reacts with 2-thiobarbituric acid under acidic conditions to form a pink complex with maximum absorbance at 532 nm, which can be extracted using an organic solvent such as butanol. The result is shown as the amount of free MDA produced, as MDA is commonly used to calibrate this test. A sample (40 µL from testes, epididymis, and hypothalamus) was mixed with 50 µL of 30% TCA in 160 mL Tris-KCl buffer. After adding 50 µL of 0.75% TBA, the mixture was incubated for 45 min at 80 °C, cooled to 25 °C, and centrifuged at 3000 g for 10 min. The absorbance of 200 µL supernatant was measured against a distilled water blank at 532 nm using a microplate reader. The method of Bergmeyer et al. [34] was used to assess the activity of xanthine oxidase (XO) in the testes, epididymis and hypothalamus. Uric acid is produced from xanthine by XO, and its absorbance at 290 nm is used in the assay. A volume of 8 µL of the sample, 150 µL of phosphate buffer, and 80 µL of xanthine solution was accurately pipetted into a microplate. Following mixing, absorbance (290 nm) was measured at one-minute intervals over a three-minute period. Measurements were taken relative to a blank prepared by replacing 8 µL of the sample with distilled water.

S 2.4. Evaluation of Apoptosis Biomarkers

The concentrations of Bcl-2 Associated X Protein (Bax), protein 53 (TP53), and B-cell Lymphoma 2 (Bcl-2) were measured from the supernatant of the tissue homogenate using ELISA, according to the manufacturer's instructions. Seven microplate wells were each filled with

100 µL of the standard working solution, while the rest were filled with 100 µL of homogenates from testes, epididymides, and hypothalamus. The plate was sealed and incubated at 37 °C for 90 minutes. After draining the wells, 100 µL of biotinylated detection antibody solution was added and the plate was mixed, resealed, and incubated for another hour at 37 °C. Wells were washed with 350 µL buffer, soaked briefly, and dried. Next, 90 µL of substrate reagent was added in the dark, the plate was sealed and incubated for 15 minutes at 37 °C. A plate reader (was used to measure the optical density at 450 nm after the reaction was stopped by adding 50 µL of stop solution to each well. The intensity of the colour developed in the samples is proportional to the levels of Bax, Bcl-2, and P53 present.

S 2.5. Histopathological Examination of the Testes, Epididymides and Hypothalamus

Histopathological analysis of the testes, epididymides, and hypothalamus was conducted using standard and advanced histology tissue processing and microscopy techniques [37, 38]. After euthanasia the hypothalamus was harvested and treated with 10% formalin solution, while the harvested epididymides and testes (sliced through to enhance rapid penetration of the fixative) were quickly fixed in Bouin's solution. All assed organs were allowed to soak between 72 hours and a week before subsequent processing. Following the dehydration processes, the sections were paraffin-embedded. Tissue specimens were sectioned into 4–5 µm slices using a microtome, subsequently mounted on charged slides, and stained with standard hematoxylin and eosin. Coded slides were evaluated under a Carl Zeiss Axio light microscope. For documentation, a pathologist—blinded to the treatment groups—captured representative images using a Zeiss Axiocam 512 camera attached to the microscope during the assessment.

S 2.6. Molecular Docking method

Molecular docking was conducted to assess the interaction between TQ and PPAR-α or PPAR /β. The molecular docking scores, reflecting the binding affinity between the ligand and the proteins, were quantified as binding constants (Kd). To achieve this, the 3-D structures of TQ were sourced from PubChem: https://pubchem.ncbi.nlm.nih.gov, while the structures of the PPAR-α (PDB: 1I7G) and PPAR- /β (PDB: 3D5F) were acquired from the Protein Database (PDB): https://www.rcsb.org/. Protein preparation was done using UCSF ChimeraX [39]. The ligand (TQ) from the synthesised receptors was loaded into PyRx, and Open Babel was used for energy minimisation. Molecular docking was carried out using grid boxes configured according to the position of the co-crystallised ligand. The docking modes with the lowest Gibbs free energy were visualised in PyMol. The binding constant (Kd) was determined using the equation: $G = -RT \ln Kd$, where R represents the gas constant (0.001987 kcal/mol/K), T denotes the temperature in Kelvin (298K), and G refers to the Gibbs free energy.
